# Measurement invariance of the Patient Health Questionnaire (PHQ-9) and Generalized Anxiety Disorder scale (GAD-7) across four European countries during the COVID-19 pandemic

**DOI:** 10.1186/s12888-022-03787-5

**Published:** 2022-03-01

**Authors:** Mark Shevlin, Sarah Butter, Orla McBride, Jamie Murphy, Jilly Gibson-Miller, Todd K. Hartman, Liat Levita, Liam Mason, Anton P. Martinez, Ryan McKay, Thomas VA Stocks, Kate M Bennett, Philip Hyland, Frédérique Vallieres, Carmen Valiente, Carmelo Vazquez, Alba Contreras, Vanesa Peinado, Almudena Trucharte, Marco Bertamini, Anna Panzeri, Giovanni Bruno, Umberto Granziol, Giuseppe Mignemi, Andrea Spoto, Giulio Vidotto, Richard P. Bentall

**Affiliations:** 1 Ulster University, Coleraine, Northern Ireland; 2 Department of Psychology, University of Sheffield, Cathedral Court, 1 Vicar Lane, S1 2LT Sheffield, England; 3 University of Manchester, Manchester, England; 4 University College London, London, England; 5 Royal Holloway, University of London, Egham, England; 6 University of Liverpool, Liverpool, England; 7 Maynooth University, Maynooth, Ireland; 8 Trinity College Dublin, Dublin, Ireland; 9 Complutense University of Madrid, Madrid, Spain; 10 University of Padua, Padua, Italy

**Keywords:** PHQ-9, GAD-7, Depression, Anxiety, Measurement invariance

## Abstract

**Background:**

The Patient Health Questionnaire (PHQ-9) and Generalized Anxiety Disorder scale (GAD-7) are self-report measures of major depressive disorder and generalised anxiety disorder. The primary aim of this study was to test for differential item functioning (DIF) on the PHQ-9 and GAD-7 items based on age, sex (males and females), and country.

**Method:**

Data from nationally representative surveys in UK, Ireland, Spain, and Italy (combined *N* = 6,054) were used to fit confirmatory factor analytic and multiple-indictor multiple-causes models.

**Results:**

Spain and Italy had higher latent variable means than the UK and Ireland for both anxiety and depression, but there was no evidence for differential items functioning.

**Conclusions:**

The PHQ-9 and GAD-7 scores were found to be unidimensional, reliable, and largely free of DIF in data from four large nationally representative samples of the general population in the UK, Ireland, Italy and Spain.

**Supplementary Information:**

The online version contains supplementary material available at 10.1186/s12888-022-03787-5.

## Background

The Patient Health Questionnaire (PHQ-9) [1] and Generalized Anxiety Disorder scale (GAD-7) [2] are widely used self-report measures of major depressive disorder (MDD) and generalised anxiety disorder (GAD). They were designed to provide an assessment of depression and anxiety symptom severity, as well as the identification of probable diagnostic cases of MDD and GAD. These measures are routinely used in primary and secondary care settings [3], as primary outcome measures in psychological treatment studies [4], and in large epidemiological surveys [5, 6].

The PHQ-9 and GAD-7 have also been used extensively in COVID-19 related research. The main longitudinal studies in the United Kingdom (UK), for example, use both measures as primary indicators of population mental health and changes over time [7, 8]. Findings from the early period of the pandemic indicated that there were significant sex and age differences, with females and younger people scoring significantly higher on the PHQ-9 and GAD-7 [9, 10], and there is also some evidence of variation in the estimated prevalence rates of depression and generalised anxiety disorder across countries, including European countries [11]. The ability to make valid sex, age, and country comparisons of depression and anxiety scores (derived from the PHQ-9 and GAD-7) is predicated on the assumption that the items contained within these scales operate equivalently for these different groups of interest. This assumption is also known as ‘measurement invariance’ [12].

Teymoori, Real, et al. [13] noted that, despite their widespread use, there was a dearth of studies that have evaluated the measurement invariance of the PHQ-9 and GAD-7. Using data from a European-wide (8 countries) sample of patients after traumatic brain injury and three different methods to detect invariance, they found that the scale items performed equally well across groups based on gender, patient type, and linguistic background. This adds to the research that has shown invariance of GAD-7 scores based on gender in a clinical sample [14] and age, gender, education, and urbanicity in Chinese medical students [15]. Similarly, PHQ-9 scores have been shown to be invariant across age, gender, race/ethnicity, marital status, education level, and health conditions [16] and time [17]. These studies have used a variety of techniques to assess invariance.

One statistical approach to assess measurement invariance is to test for the presence of differential item functioning (DIF) [18]. DIF is assessed by identifying if there are differences in individual item scores across groups (e.g., sex, countries) or levels of a variable (e.g., age) whilst controlling for the overall construct (latent variable) being measured. Within the literature there are different statistical methods to assess DIF, each with their own advantages and disadvantages (see [19, 20] for reviews). In this study, we opted to use the multiple indicators multiple causes (MIMIC) approach [21, 22] as it (1) allows the specification and estimation of a multidimensional latent variable model with the grouping variables, (2) provides a range of absolute and relative fit statistics, (3) employs maximum likelihood estimation to deal with non-normality, and (4) has greater statistical power than multiple-group models.

The primary objective in this study, therefore, was to test for DIF on the PHQ-9 and GAD-7 items based on age, sex (males and females), and country (UK, Ireland, Spain, and Italy). This study adds to the extant research literature as there is a relative dearth of invariance studies based on large community or nationally representative population-based samples. In addition, many previous studies have analysed either the GAD-7 or the PHQ-9 alone, or tested invariance for the two scales separately; our study tests for invariance using a combined two-factor model using the GAD-7 and the PHQ-9 items in the same model. Finally, data collection for this study was conducted early in the COVID-19 pandemic when the population levels of anxiety and depression were likely to be elevated [10] thereby capturing a wide range of scores on the GAD-7 and the PHQ-9.

## Method

### Participants and Procedure

The COVID-19 Psychological Research Consortium (C19PRC) comprises researchers from the UK, Ireland, Spain, and Italy. The Consortium was established in March 2020 with the aim of monitoring the psychological response to the emerging COVID-19 pandemic. Data from the four European countries were used in this study as these surveys were similar and comparable in terms of the sampling strategy employed.

In the UK, the first wave of data collection started on 23rd March 2020 and was completed on 28th March 2020. Participants (*N* = 2,025) were recruited from an online research panel (*Qualtrics*) using stratified quota sampling to ensure that the sample characteristics of sex, age, and household income (quintiles) were representative of the UK adult population. Participants were recruited from the four countries of the UK and an approximately representative distribution was achieved relative to population size: England (83.7%), Wales (3.0%), Scotland (7.5%), and Northern Ireland (2.2%), while 3.7% did not provide their postcode stem for their residency status to be determined. The mean age of the sample was 45.44 years (*SD* = 15.90), and 51.7% (*n* = 1047) were female, 48.0% male (*n* = 972) and 0.3% (*n* = 6) checked the transgender/prefer not to say/other option. The majority of the sample were born in the UK (90.6%, *n* = 1834). The ethnic profile of respondents was diverse and closely mirrored that of the UK population: White British/Irish (*n* = 1732, 85.5%), White non-British/Irish (*n* = 116, 5.7%), Indian (*n* = 41, 2.0%), Pakistani (*n* = 27, 1.3%), Chinese (*n* = 19, 0.9%), Afro-Caribbean (*n* = 13, 0.6%), African (*n* = 27, 1.3%), Arab (*n* = 3, 0.1%), Bangladeshi (*n* = 6, 0.3%), other Asian (*n* = 11, 0.5%) and other ethnic group (*n* = 30, 1.5%). Nearly half of the respondents were in full-time employment (48.8%, *n* = 988), 15.0% (*n* = 303) were in part-time employment, 16.5% (*n* = 334) were retired, 4.7% (*n* = 95) were students, and 5.1% (*n* = 103) were currently unemployed and seeking work, 3.4% (*n* = 69) were not working due to disability, and 6.6% (*n* = 133) were unemployed and not seeking work. Full details of the methods employed and information on the representativeness of the sample have previously been reported [8]. Ethical approval for the UK survey was granted by the University of Sheffield (Reference number 033759).

In Ireland, participants (*N* = 1,041) were also recruited online via *Qualtrics*, using stratified, quota sampling to select participants that were representative of the general adult population of the Republic of Ireland (ROI) in relation to sex, age and geographical location (i.e. from the four provinces of the ROI: Leinster, Munster, Connacht and Ulster). Wave 1 data was collected between 31st March 2020 – 5th April 2020. The mean age of the Irish sample was 44.97 years (*SD* = 15.76) and 51.5% (*n* = 536) were female, 48.2% (*n* = 502) were male and the remaining 0.3% (*n* = 3) reported being another gender or preferred not to say. Residency was representative across the four provinces of the ROI; Leinster (*n* = 576, 55.3%), Munster (*n* = 284; 27.3%), Connacht (*n* = 125; 12.0%) and part of Ulster (*n* = 56; 5.4%). Over two-thirds of the sample were born in Ireland (*n* = 736, 70.7%) and three-quarters reporting being of Irish ethnicity (*n* = 779; 74.8%). At the time of the Wave 1 survey, 43.3% of the sample reported being employed fulltime (including self-employed, *n* = 451) and a further 15.7% were employed parttime (including self-employed, *n* = 163). The remainder of the sample was made up of retirees (*n* = 156, 15.0%), those recently unemployed due to the pandemic (*n* = 59, 5.7%), those unemployed not due to the pandemic (*n* = 88, 8.5%), students (*n* = 66, 6.3%) and those that cannot work due to disability, illness or another reason (*n* = 58, 5.6%). Ethical approval was obtained by the Social Research Ethics Committee at Maynooth University [Ref SRESC-2020-2402202]. Full details of the Irish survey have previously been reported [23].

In Spain, (*N* = 1,949) adults (18+ years) were recruited by Sondea, a company that provides online samples for research, based on a large national panel of participants. Quota sampling was used to ensure that the sample was representative of the Spanish adult population in relation to sex, age and political region. Participants completed the survey online in Spanish via the Qualtrics survey platform between the 7th – 14th April 2020. The average age of the sample was 45.17 years (*SD* = 12.77) and 52.7% were male (*n* = 1,027), 47.0% were female (*n* = 917) and 0.3% (*n* = 5) reported another gender classification. Residence was representative across the 19 autonomous provinces and cities; Andalusia (*n =* 356, 18.3%), Aragon (*n =* 47, 2.4%), Asturias (*n =* 46, 2.4%), Balearic Islands (*n =* 27, 1.4%), Canary Islands (*n =* 79, 4.1%), Cantabria (*n =* 31, 1.6%), Castile-La Mancha (*n =* 66, 3.4%), Castile and Leon (*n =* 122, 6.3%), Catalonia (*n =* 266, 13.6%), Ceuta (*n =* 3, 0.2%), Extremadura (*n =* 54, 2.8%), Galicia (*n =* 145, 7.4%), Madrid (*n =* 291, 14.9%), Melilla (*n =*3, 0.2%), Region of Murcia (*n =* 61, 3.1%), Navarra (*n =* 35, 1.8%), Basque Country (*n =* 85, 4.4%), La Rioja (*n =* 16, 0.8%) and Valencian Community (*n =* 215, 11.0%). The majority of respondents were born in Spain (*n* = 1812; 93.0%) and lived in an urban area (*n* = 1642, 84.2%). Over half (57.5%) of the sample reported being employed full-time, 10.0% part-time and 17.1% were unemployed. The rest of the sample was made up with retirees (8.7%), students (5.6%) and those that cannot work due to disability or other reason (0.9%). Ethical approval for the study was obtained from the School of Psychology (Complutense University Madrid) Deontological Commission (Ref: 2019/20-034). Full details of the survey method and procedures have previously been reported [24].

In the Italian study, the survey was administered in four regions – Campania, Lazio, Lombardia and Veneto. These regions were selected as they represented the northern (Lombardia, Veneto), central (Lazio), and southern (Campania) parts of the country. They are also large regions in terms of populations (the four regions cover almost half of the total Italian population), and provided variation in terms of Covid-19 infection rates (highest in Lombardia, and very low in Campania). Quota sampling was used to ensure that the sample characteristics of gender, age, household income, and region (Campania, Lazio, Lombardia, and Veneto) matched the Italian population. Participants were required to be an adult (18 years or older) and a resident in one of these regions. Participants completed the survey online from 13th - 28th July 2020. Participants were recruited via *Qualtrics* and completed the survey online. In total, *N* = 1,048 valid respondents were recruited, however, for the purposes of the current study, a small number of cases with missing data on the PHQ-9 and GAD-7 were removed, resulting in a final sample of *N* = 1039. The mean age was 49.94 years (*SD* = 16.14) and, 51.2% (*n* = 532) were female and 48.8% (*n* = 507) were male. Participants were recruited from the four selected regions based on their population size: Campania (*n* = 227), Lazio (*n* = 234), Lombardia (*n* = 392), Veneto (*n* = 186). The vast majority of participants reported Italian nationality (*n* = 1,004; 96.6%) and Caucasic ethnicity (*n* = 775, 74.7%). The sample mainly consisted of 461 full-time employed (44.4%), 251 retired (24.2%) and 112 participants who were unemployed or looking for work (10.8%). Ethical approval for this study was provided by the Ethical Committee for Psychological Research of the University of Padua (protocol: 3818). Further details of the sample and methodology have previously been reported [25].

## Measures

Age (measured in years), sex (0 = male, 1 = female), and country were used as variables to detect possible DIF. The four countries were dummy coded using UK as the reference category.

Depression and anxiety: In all surveys, depression was measured using the *Patient Health Questionnaire-9* (PHQ-9) [1] and anxiety was measured using the *Generalized Anxiety Disorder 7-item Scale* (GAD-7) [2]. Both scales instruct participants to indicate how often they have been bothered by each symptom over the last two weeks using a four-point Likert scale ranging from 0 (*Not at all*) to 3 (*Nearly every day*). Examples items are “Feeling down, depressed or hopeless” (PHQ) and “Not being able to stop or control worrying?” (GAD). Possible scores on the PHQ-9 range from 0 to 27, and on the GAD-7 from 0 to 21, with higher scores indicating higher levels of depression and anxiety. Scale scores of 10 or greater are typically used to indicate probable diagnostic status on each of these measures [1, 2]. The psychometric properties of the PHQ-9 [26] and GAD-7 [27] scores have been widely supported in other studies. In each country, the internal reliability estimates, as assessed by Cronbach’s alpha (α), of the PHQ-9 scores (UK α = 0.921; Ireland α = 0.905; Spain α = 0.889; Italy α = 0.918) and the GAD-7 scores (UK α = 0.921; Ireland α = 0.905; Spain α = 0.889; Italy α = 0.918) were high. Language specific versions of the scales were used [28].

### Statistical Analysis

The analyses were conducted in three phases. First, descriptive statistics for the summed scores on the PHQ-9 and GAD-7 were calculated and cross-country differences were tested using ANOVA. Second, a confirmatory factor analysis (CFA) model of the PHQ-9 and GAD-7 indicators was estimated to establish the fit of a baseline model for each of the four countries. The model specified two correlated latent variables, with the PHQ-9 item loading on a ‘Depression’ latent variable and the GAD-7 items loading on an ‘Anxiety’ latent variable. The data from the four countries were then combined and tests of configural and metric invariance were conducted: configural invariance tests that the latent structure (i.e., a correlated two-factor model) is consistent across the groups, and metric invariance adds constraints to test for the equality of factor loadings across the groups. Scalar invariance was not tested as differences in the intercepts were assessed as part of the DIF analysis.

Third, a MIMIC model was specified to test for DIF on the PHQ-9/GAD-7 items based on the exogenous predictor variables of country, age, and sex. The MIMIC models provides information on:

1. the factor loadings for the PHQ-9/GAD-7 measurement model;
2. the relationships between the predictor variables and the latent variables (these regression coefficients indicate mean differences at the level of the latent variable across different levels of the predictor variables); and
3. direct effects between the predictor variables and the PHQ-9/GAD-7 items, independent of variability on the latent variables. Any significant direct effects are indicative of DIF

The MIMIC model was initially specified with only dummy-coded variables to indicate country to determine the magnitude and significance of any cross-country differences in the mean level of anxiety and depression. A subsequent model also included the age and sex variables and the process of establishing DIF was conducted.

To determine which direct effects should be included, modification indices (MIs) [29] and the standardised expected parameter change (SEPCs) [30, 31] values were used. MIs indicate which path could be added to the model that would significantly improve model fit if freely estimated, that is, reduce the chi-square by 3.84 or more (which is the critical value for the chi-square for one degree of freedom, *p* < .05). In practice, a more conservative value of 10 was used to ensure that small inconsequential parameters were not added, and this is reflected in Mplus only reporting MIs greater than 10. The SEPC indicates the estimated value of a fixed parameter (in this case fixed to zero) if it were estimated, that is, the expected standardised regression coefficient. The MIs are influenced by sample size [32], and with a very large sample this is likely to indicate that parameters with very small absolute values should be added to the model. On this basis, Kaplan [33] proposed that a combination of MIs and SEPCs should be used to determine which parameters should be added to the model. Thus, in this study, a direct effect from the predictor to a PHQ-9/GAD-7 item would be added if the MI was greater than 10 *and* the SEPC was greater than 0.20. A process followed whereby the path with the largest MI/SEPC was freely estimated in the model and the model was re-estimated. This continued until there were no MIs/SEPCs greater than 10/0.20. All analyses were conducted in Mplus 8.1 [34].

The model parameters were estimated using robust maximum likelihood estimation (MLR) [35], and a range of fit statistics were used to assess the goodness of fit for each model: the Chi-square, the comparative fit index (CFI) [36], and the Tucker-Lewis Index (TLI) [37]. A non-significant chi-square and values greater than 0.90 for the CFI and TLI were considered to reflect acceptable model fit. Additionally, the Root Mean Square Error of Approximation (RMSEA) [38] was reported, where a value less than 0.05 indicated close fit and values up to 0.08 indicated reasonable errors of approximation. The same cut-off values can be used for the standardized root mean square residual (SRMR) [39]. To compare the configural and metric models of invariance the criteria proposed by Chen [40] were used: less than 0.010 change in CFI, less than 0.015 in RMSEA, and less than 0.030 for the SRMR.

## Results

The mean GAD-7 and PHQ-9 scores across the countries are reported in Table 1.

**Table 1:** Descriptive Statistics for the GAD-7 and PHQ-9 Scores

	UK^a^	Ireland^b^	Spain^c^	Italy^d^	ANOVA	Contrasts	h^2^
Mean (*SD*) GAD-7	5.15 (5.68)	5.03 (5.52)	5.86 (5.24)	5.73 (5.14)	F (3, 6050) = 8.80, *p* < .001	a, b < c, d	0.004
Mean (*SD*) PHQ-9	5.37 (6.21)	5.78 (6.09)	6.50 (5.65)	6.68 (5.84)	F (3, 6050) = 17.01, *p* < .001	a, b < c, d	0.008

Spain and Italy had higher mean scores than the UK and Ireland for both anxiety and depression, and a one-way ANOVA indicated that all the means were not equal^1^. Post-hoc pairwise Bonferroni tests indicated that that there were no significant differences in anxiety scores between the UK and Ireland (*p* = 1.00), and between Spain and Italy (*p* = 1.00). Anxiety scores in the UK were significantly lower than Spain (*p* < .001) and Italy (*p* < .05), and anxiety scores in Ireland were also significantly lower than Spain (*p* < .001) and Italy (*p* < .05). The pattern of differences (and significance) was the same for depression scores. The effect sizes for both anxiety (h^2^ = 0.004) and depression (h^2^ = 0.008) were very small.

The CFA fit statistics in Table 2 show that the correlated two-factor model was acceptable in each national sample on all fit statistics except the chi-square. The chi-square was significant for all models: however, this should not lead to rejection of these models as the power of chi-square tests is positively related to sample size [41]. The standardised factor loadings were all positive, high, and statistically significant (*p* < .001), and these are reported in Table S1 in the Supplementary Materials. The configural and metric models of invariance also indicted adequate model fit based on the differences in the CFI, RMSEA and SRMR (ΔCFI = -0.003, ΔRMSEA = 0.001, ΔSRMR = 0.017).

**Table 2:** Fit Statistics for the Correlated Two-Factor Model and Tests of Invariance for the GAD-7 and PHQ-9 Items

Model	*χ* ^2^	df	*p*	CFI	TLI	RMSEA	SRMR
CFA							
UK	1,019.749	103	< 0.001	0.933	0.921	0.066 (0.063, 0.070)	0.042
Ireland	580.696	103	< 0.001	0.930	0.918	0.067 (0.062, 0.072)	0.043
Spain	1,079.059	103	< 0.001	0.928	0.916	0.070 (0.066, 0.074)	0.041
Italy	495.612	103	< 0.001	0.949	0.941	0.061 (0.055, 0.066)	0.034
Invariance							
Configural	3,187.083	412	< 0.001	0.933	0.922	0.067 (0.065, 0.069)	0.040
Metric	3,639.737	454	< 0.001	0.924	0.919	0.068 (0.066, 0.070)	0.057

The data from the four countries were combined and dummy coded country variables were added to the model with the UK as the reference category. The standardised regression coefficients from the country variables to the depression latent variable indicated that there was no significant difference in the factor means for the UK and Ireland (β = 0.023, *p* = .138), but higher means for Spain (β = 0.090, *p* < .001) and Italy (β = 0.082, *p* < .001); the effect sizes were small, accounting for less than 1% of the variance in the depression latent variable (R-square = 0.009, *p* < .001). Similarly, the standardised regression coefficients for the anxiety latent variable indicated that there was no significant difference in the factor means for the UK and Ireland (β = -0.009, *p* = .556), but higher means for Spain (β = 0.069, *p* < .001) and Italy (β = 0.046, *p* < .01), accounting a very small proportion of the variance in the anxiety latent variable (R-square = 0.006, *p* < .01).

The age and gender variables were added to the model as predictors of the depression and anxiety latent variables, and the standardised regression coefficients are reported in Table 3.

**Table 3:** Standardised Regression Coefficients For Predictors of Depression and Anxiety Latent Variables

Predictor	Latent variable	
	Depression	Anxiety
Age (years)	-0.302^***^	-0.255^***^
Sex (female)	0.083^***^	0.128^***^
Ireland	0.021	-0.011
Spain	0.092^***^	0.073^***^
Italy	0.117^***^	0.075^***^
R-square	0.114^***^	0.096^***^

^***^p < .001

Depression and anxiety were both negatively associated with age, and the coefficients for sex indicated significantly higher levels of anxiety and depression for females. The coefficients for the dummy-coded country variables indicated significantly higher levels of depression and anxiety for Spain and Italy compared to the UK, and no difference to Ireland while adjusting for age and gender. The sex, age, and country variables explained 11.4% and 9.6% of the variance in the depression and anxiety latent variables, respectively.

The largest MI and SEPC was a direct effect between the variable representing Spain and the second GAD item (‘*Not being able to stop or control worrying*’: MI = 375.736, SEPC = 0.174). This direct effect was added, and the model was re-estimated. The next largest MI/SEPC was a direct effect between the variable representing Spain and the ninth PHQ item (‘*Thoughts that you would be better off dead or of hurting yourself in some way*’: MI = 145.819, SEPC = -0.149). When this direct effect was added to the model and re-estimated, the next largest MI/SEPC was a direct effect between sex and the ninth PHQ item (MI = 78.000, SEPC = -0.108). When this effect was added and the model was re-estimated, there were no other direct effects to be included based on the MI/SEPC inclusion criterion.

The final model estimates show that the three direct effects were small in magnitude (Sex -> PHQ item 9 = -0.108, *p* < .001; Spain -> PHQ item 9 = -0.155, *p* < .001; Spain -> GAD item 2 = 0.174, *p* < .001); furthermore, the difference in the R-square for the two items before and after the inclusion of the direct effects was small. For GAD item 2 the R-square increased from 0.703 to 0.732, so the DIF accounted for 2.9% of the variance in that item, and for PHQ item 9 the R-square increased from 0.379 to 0.414, so the DIF accounted for 3.5% of the variance in that item. The fit statistics for the DIF model of depression and anxiety are reported in Table 4.

**Table 4:** Fit Statistics for the DIF Model of Depression and Anxiety

Model	*χ* ^2^	df	*p*	CFI	TLI	RMSEA	SRMR
Baseline	4,464.357	173	< 0.001	0.915	0.901	0.064 (0.062, 0.066)	0.039
Spain -> GAD 2	4,067.576	172	< 0.001	0.923	0.910	0.061 (0.060, 0.063)	0.037
Spain -> PHQ 9	3,911.610	171	< 0.001	0.926	0.913	0.060 (0.059, 0.062)	0.036
Gender -> PHQ 9	3,825.079	170	< 0.001	0.927	0.915	0.060 (0.058, 0.061)	0.035

## Discussion

The primary objective in this study was to test for DIF on the PHQ-9 and GAD-7 items based on age, sex (males and females), and country (UK, Ireland, Spain, and Italy). In all countries quota sampling was used to collect data that was representative of the populations on benchmarked demographic variables. Initial CFA analyses indicated a model with two correlated latent variables - the PHQ-9 items loaded on a ‘Depression’ latent variable, and the GAD-7 items loaded on an ‘Anxiety’ latent variable - was an acceptable description of the data. There were country differences on the summed scales, with UK and Ireland scoring significantly lower than Spain and Italy, though the effect size was very small.

Initial analyses indicated that the PHQ-9 and GAD-7 items were good indictors of the depression and anxiety latent variables, respectively. For all countries, the factor loadings were high, positive and statistically significant. The estimates of internal consistency were high for both scales for all countries. These positive psychometric characteristics of the PHQ-9 and GAD-7 scale scores have been reported previously [16].

The DIF analysis indicated that after controlling for the overall level of depression, females and participants from Spain (compared to UK) scored lower on the ninth PHQ item (‘*Thoughts that you would be better off dead or of hurting yourself in some way*’); however, the size of these effects were small and would not be likely to contribute to incorrect conclusions about group differences on the PHQ-9 scale scores. Similarly, after controlling for the overall level of anxiety the participants from Spain (compared to UK) scored higher on the second GAD item (‘*Not being able to stop or control worrying*’); again, the size of this effect was small and unlikely to contribute to problematic DIF. Overall, these findings support the use of PHQ-9 and GAD-7 in the general population to make comparisons based on age, gender and country. Our findings are consistent with a recent systematic review of 10 invariance studies, largely among clinical samples, of the PHQ-9 that concluded that the results “…*established measurement invariance of the PHQ-9 across sociodemographic variables*” (p.223) [16], and a comprehensive analysis of the GAD-7 concluded that the scores were “…*invariant across sociodemographic groups and over time*” [42]. The findings from our study add to the extant research literature on the PHQ-9 and GAD-7 by indicating measurement invariance in large nationally representative sample of four European countries taken during a global pandemic.

The findings from this study should be considered in light of some limitations. First, not all surveys were conducted at the same time, the survey in Italy took place about 3 months later than the others, and so some mean differences between countries may reflect this. Second, the data were collected at one time point, and so the invariance of the scores across time could not be assessed. Finally, these analyses tested for uniform DIF rather than non-uniform DIF (where the effect of the predictor variable on the item is not constant across all levels of the latent variable), but there was no *a priori* reason to expect non-uniform DIF.

## Conclusions

In conclusion, the PHQ-9 and GAD-7 scores were found to be unidimensional, reliable, and largely free of DIF in data from four large nationally representative samples of the general population in the UK, Ireland, Spain and Italy. Our findings support the use of these widely scales to make comparisons between these countries, for males and females, of all ages. This provides further support for the effectiveness of the PHQ-9 and GAD-7 as screening instruments for depression and anxiety [43]. Future research should aim to establish invariance across other countries, to ensure that valid international comparisons can be made in comparative research. This will benefit mental health professionals, epidemiologists and public health professions make informed decisions about levels of mood and anxiety disorders.

## Supplementary information

**Additional file 1:**  **Table S1.** Standardised Factor Loadings for PHQ-GAD Confirmatory Factor Analysis for Each Country.

**Additional file 2:  Table S2. **Shapiro-Wilk Normality Tests for PHQ and GAD-7 Scores for each Country

## Data Availability

The datasets used and/or analyzed during the current study is available at https://osf.io/zjnhk/.

## Abbreviations

ANOVA: Analysis of variance
C19PRC: COVID-19 Psychological Research Consortium
CFA: Confirmatory factor analysis
CFI: Comparative fit index
DIF: Differential item functioning
GAD: Generalized anxiety disorder
GAD-7: Generalized Anxiety Disorder scale
MDD: Major depressive disorder
MI: Modification index
MIMIC: Multiple indicators multiple causes
MLR: Robust maximum likelihood estimation
PHQ-9: Patient Health Questionnaire
RMSEA: Root mean square error of approximation
ROI: Republic of Ireland
SEPC: Standardised expected parameter change
SRMR: Standardized root mean square residual
TLI: Tucker-Lewis Index
UK: United Kingdom

## Footnotes

1 The PHQ and GAD scores all showed non-normal distributions (see Table S2) so we also analysed the data using a non-parametric test, the Kruskal-Wallis Test, and it confirmed the rejection of the null hypothesis of equal means (F = 129.01, df = 3, *p* < .001).

## Declarations

Sheffield (Reference number: 033759), Maynooth University (Reference number: SRESC-2020-2402202), Complutense University Madrid (Reference number: 2019/20-034), and University of Padua (Reference number: 3818). All participants gave their informed electronic consent to participate in the survey. All methods were carried out in accordance with the relevant guidelines and regulations.
