# Supplementary material for: Measurement invariance of the Patient Health Questionnaire (PHQ-9) and Generalized Anxiety Disorder scale (GAD-7) across four European countries during the COVID-19 pandemic
Source: BMC Psychiatry. 2022 Mar 1;22:154. doi: 10.1186/s12888-022-03787-5 (PMC8886334; doi:10.1186/s12888-022-03787-5)
Supplement: Supplementary file 1 — Additional file 1: Table S1. Standardised Factor Loadings for PHQ-GAD Confirmatory Factor Analysis for Each Country. [file 12888_2022_3787_MOESM1_ESM.docx]

Table S1. Standardised Factor Loadings for PHQ-GAD Confirmatory Factor Analysis for Each Country.

| Item | UK | Ireland | Spain | Italy |
| --- | --- | --- | --- | --- |
|  |  |  |  |  |
| Depression |  |  |  |  |
| PHQ 1 | .782 | .707 | .726 | .650 |
| PHQ 2 | .838 | .822 | .806 | .814 |
| PHQ 3 | .708 | .714 | .685 | .695 |
| PHQ 4 | .757 | .735 | .784 | .717 |
| PHQ 5 | .730 | .654 | .654 | .738 |
| PHQ 6 | .792 | .810 | .725 | .819 |
| PHQ 7 | .804 | .790 | .727 | .771 |
| PHQ 8 | .682 | .602 | .589 | .767 |
| PHQ 9 | .697 | .635 | .467 | .735 |
|  |  |  |  |  |
| Anxiety |  |  |  |  |
| GAD 1 | .856 | .866 | .837 | .820 |
| GAD 2 | .897 | .884 | .770 | .861 |
| GAD 3 | .900 | .880 | .848 | .861 |
| GAD 4 | .873 | .874 | .894 | .847 |
| GAD 5 | .755 | .742 | .816 | .787 |
| GAD 6 | .773 | .744 | .726 | .801 |
| GAD 7 | .833 | .807 | .732 | .799 |
|  |  |  |  |  |
| Factor correlation | .862 | .870 | .861 | .801 |

Note: All loadings and correlations significant p < .001. All cross-loadings were fixed at zero.
