# Supplementary material for: Measurement invariance of the Patient Health Questionnaire (PHQ-9) and Generalized Anxiety Disorder scale (GAD-7) across four European countries during the COVID-19 pandemic
Source: BMC Psychiatry. 2022 Mar 1;22:154. doi: 10.1186/s12888-022-03787-5 (PMC8886334; doi:10.1186/s12888-022-03787-5)
Supplement: Supplementary file 2 — Additional file 2: Table S2. Shapiro-Wilk Normality Tests for PHQ and GAD-7 Scores for each Country [file 12888_2022_3787_MOESM2_ESM.docx]

Table S2. Shapiro-Wilk Normality Tests for PHQ and GAD-7 Scores for each Country.

|  | PHQ scores | | | | GAD-7 scores | | | |
| --- | --- | --- | --- | --- | --- | --- | --- | --- |
|  | UK | Ireland | Spain | Italy | UK | Ireland | Spain | Italy |
| N | 2,025 | 1,041 | 1,949 | 1,039 | 2,025 | 1,041 | 1,949 | 1,039 |
| Shapiro-Wilk | 0.825 | 0.853 | 0.911 | 0.898 | 0.844 | 0.843 | 0.906 | 0.899 |
| p-value | <.001 | < .001 | < .001 | < .001 | < .001 | < .001 | < .001 | < .001 |
